# Predictors of modern contraceptive usage among sexually active rural women in Ethiopia: A multi-level analysis

**DOI:** 10.1186/s13690-021-00621-4

**Published:** 2021-06-04

**Authors:** Setegn Muche Fenta, Shewayiref Geremew Gebremichael

**Affiliations:** Department of Statistics, Faculty of Natural and Computational Sciences, Debre Tabor University, Debre Tabor, Ethiopia

**Keywords:** Ethiopia, Multi-level, Modern contraceptive, Rural women

## Abstract

**Background:**

Ethiopia is one of the Sub-Saharan Africa countries with the lowest modern contraceptive prevalence rate and the highest fertility rate. This study aimed to assess individual and community-level predictors of modern contraceptive use among sexually active rural women in Ethiopia.

**Data and methods:**

A sample of 9450 sexual active rural women aged 15-49 was extracted from the 15, 683 nationally representative samples of 2016 Ethiopian Demographic and Health Survey (EDHS). Multi-level logistic regression model was considered to identify determinant factors of modern contraceptive use among sexually active rural women in Ethiopia.

**Result:**

The prevalence of modern contraceptive use among respondents was 20% in rural Ethiopia. Injection (66.35%) was the most common type of modern contraceptive use. In the last full model of the multilevel analysis, individual and community-level factors accounted for 86.69% of the variation in the use of modern contraceptive methods. Secondary and above-educated women (AOR = 1.39, 95%CI: 1.06, 2.81), having 1-4 living children (AOR = 2.70, 95%CI: 2.07, 3.53), rich wealth status (AOR = 2.26, 95%CI: 1.96, 2.60), married women (AOR = 17.31, 95%CI: 10.72, 27.94), having primary educated husband (AOR = 1.45, 95%CI: 1.27, 1.67) and being working husband (AOR = 2.26, 95%CI: 1.96, 2.60) were significantly positively associated with individual-level factors of the use of modern contraceptive methods. Besides, modern contraceptive use was negatively associated with Muslim women (AOR = 0.29, 95%CI: 0.25, 0.33). Compared to the Tigray region, women living in the Afar, Somali, Harari, and Dire Dawa regions had lower use of modern contraceptive methods. Women who had access to mass media (AOR = 1.35, 95%CI: 1.16, 1.57) were more likely to use contraceptives than their counterparts.

**Conclusion:**

The prevalence of modern contraceptive use among rural women has very low. Both individual and community-level factors were significant predictors of modern contraceptive use. Consequently, the government and other stakeholders need to address educational opportunities; creating awareness about modern contraception and valuable counseling would increase modern contraceptive methods utilization.

## Background

In mid-2019, the world population had reached 7.7 billion, adding one billion people since 2007 and two billion since 1994. In 2030, the world population estimate will be 8.5 billion; in 2050, 9.7 billion, and 10.9 billion in 2100. Over the coming decades, Sub-Saharan Africa will account for most of the growth of the world’s population, while many regions will begin to experience declining numbers of the population. About two billion people might be added to the global population between 2019 and 2050, 1.05 billion (52%) might be added in countries of sub-Saharan Africa. The result of the high fertility rate is poor health conditions in general, and inadequate availability of medical care, the risks of pregnancy are higher in Africa than anywhere else [1, 2].

With a current population of 112 million with a fertility rate of 4.6 children born per woman, Ethiopia leads sub-Saharan Africa; one of the highest populations in Africa [1–7]. High fertility carries the highest risk of child mortality, as it is generally difficult for Ethiopian families to provide adequate food and health care for all their children. Hence, children could be easily affected by severe malnutrition and infections. In addition, the problem of overpopulation has also been compounded by poverty, war, drought, inadequate infrastructure, and poor agricultural and industrial production [8, 9].

In 2019, 190 million women of reproductive age worldwide who want to avoid pregnancy do not use any contraceptive method. About 43% of the 206 million pregnancies that occurred in the developing world in 2017 would be unintended. Over 80% of women infected with common, curable sexually transmitted infections in developing countries are not treated [10–13]. Modern contraceptives improve the rights of people to decide their children’s number and gap [14]. By avoiding unintended and high-risk pregnancies and reducing the need for unsafe abortions contraceptive use reduces maternal mortality and improves women’s health. Some contraceptives also enhance the health of women by reducing the risk of transmitting disease and protecting them from certain cancers and health problems [15, 16]. The use of contraceptives has increased in many parts of the world, especially in Asia and Latin America, but in sub-Saharan Africa, continue to decline. The use of modern contraceptives has slightly increased worldwide, from 54% in 1990 to 57.4% in 2015. It goes from 23.6 to 28.5% in Africa [17].

Modern contraceptive use among Ethiopian married women has increased from 6% in 2000 to 38% in 2018 [5, 7, 10]. By 2020, the Ethiopian government should aim to increase the prevalence of contraception to 40% for 15 to 19 years-old women, and to 43% for 20 to 24 years-old women. Furthermore, the government is committed to reducing the unmet need for the two age groups to 10% overall [5, 11]. Nevertheless, it is still a challenge to make sure consistency in reproductive health supplies and distribution to the last mile. Gains are also precarious, with 10% more women in urban settings using modern contraception than those in rural areas. There is low contraceptive uptake and demand generation among rural populations and there is inadequate training for professionals in public health. Professionals such as-nurses and midwives may not always be ready or qualified to insert or remove techniques, including implants, and insertion kits may not even are available for the services needed. Several factors can act as obstacles to accessing family planning services for women in Ethiopia, including illiteracy, early childbearing, gender-based disparities, and religious and traditional influences. Ethiopia is far from completely addressing its population’s health needs [11, 12]. The country is unlikely to meet its Family Planning 2020 (FP2020) targets of adding 6.2 million new modern users of contraceptives by 2020 [12].

In Ethiopia, studies were conducted to identify predictors of modern contraceptive use among sexually active women [10–20]. Those studies were done by using an ordinary regression model. The ordinary logistic regression model was taking into account only individual-level factors by omitting the community (cluster) effect. Yet, its underlying assumption of individual observation must be independent each other limits its use in many real-world applications with hierarchical or correlated nature of data. For this reason, the result ordinary logistic regression model might be applicable in incorrect inference about parameter estimates, standard errors, tests, and confidence intervals. The variation in ﻿the﻿ determinants﻿ of modern contraceptive use ﻿might be﻿ due﻿ to ﻿heterogeneity ﻿in﻿ the enumeration area of﻿ the study. To address this, we proposed a multilevel logistic regression model for modern contraceptive use data [21, 22]. Therefore, this study aimed to address individual and community-level predictors of modern contraceptive use among sexually active rural women in Ethiopia.

##  Methods

### Study setting

This study was conducted in Ethiopia. Ethiopia is the second-most populous country in Africa next to Nigeria. It has a unique cultural heritage with a diverse population mix of ethnicity and religion. Located in the northeastern part of Africa; also known as the Horn of Africa. It borders six countries Eritrea (North), Djibouti (East), Somalia (South East), Kenya (South and North West), South Sudan (West), and Sudan (North West and West) [10, 11].

### Data source and design

The data used for this study was taken from the 2016 Ethiopian Demography and Health Survey (EDHS). In the 2016 EDHS, a two-stage stratified cluster sampling technique has been employed. In the first stage, enumeration areas were selected. Enumeration area is a geographic area consisting of a convenient number of dwelling units which served as a counting unit for the census. In the second stage, 28 households per enumeration area were selected with an equal chance of systematic selection per enumeration area.

### Variable of the study

#### Outcome variable

The outcome variable was modern contraceptive use among sexually active women. It is a categorical variable (Yes, No). The modern contraception method includes the pill, IUD, injections, the diaphragm, female or male sterilization, the male or female condom, implants, and lactation amenorrhea contraception method.

#### Independent variables

The independent was selected by reviews of previous literature [10–21]. The independent variables included in the study were the wealth index, educational level of the mother, educational level of husband, age of women, occupation of women, religion, age at first birth, contraceptive use, number of living children, birth in the last three years, marital status, occupation of husband, terminated pregnancy, the region they live in, and access to mass-media.

### Data management and analysis

Data were extracted and decoded using SPSS software version 21 and the decoded data were analyzed using STATA version 14. Descriptive statistics such as frequencies, percentages, and bar charts were performed to describe the study participants.

The dataset was constructed by hierarchical/multilevel study design. In the hierarchical/multilevel study design, the individual observations are not considered as independent of each other. In this study, the respondents/women are nested with Enumeration Areas (EAs). In this case, the conventional regression model is not appropriate. Having this reason, a multilevel logistic regression model was used to identify the predictors of modern contraceptive usage among sexually active rural women in Ethiopia.

In the multilevel analysis, four consecutive models were fitted [10–13]. The first is the null model (Model I), for detecting the existence of possible contextual effect which is fitted without any explanatory variable at the individual level as well as at the community level. The second model fitted by including all individual-level variables (model II). In this step, we assess the contribution of each individual-level explanatory variable, the significance of each predictor, and what changes occur in the first-level and second-level variance terms. The third model fitted by including all community-level variables (Model III). This model allows us to look at whether the community-level explanatory variables explain the between-group variation in the dependent variable.

The result of the fixed effect is reported in terms of adjusted odds ratio with 95%CI. All variables with *p* values < 0.05 have been considered as statistically significant. The measures of variation (random effects) were summarized using ICC, Median Odds Ratio (MOR), and proportional change in variance (PCV). ICC is a measure of within-cluster variation, the variation between individuals within the same cluster, and it was calculated using the formula:

$$ ICC=\frac{V_A}{V_A+\raisebox{1ex}{${\pi}^2$}\!\left/ \!\raisebox{-1ex}{$3$}\right.}=\frac{V_A}{V_A+3.29}, $$

Where; *V*_*A*_ is the estimated variance in each model, which was described elsewhere [10].

The total variation attributed to individual or/and community-level factors at each model were measured by the proportional change in variance (PCV), which is calculated as:

$$ PCV=\frac{V_A-{V}_B}{V_A}, $$

Where; *V*_*A*_ = variance of the first model, and *V*_*B*_ = variance of the model with more terms [10]. The MOR is the median odds ratio between the individual of higher propensity and the individual of lower propensity when comparing two individuals from two different randomly chosen clusters and it measures the unexplained cluster heterogeneity, the variation between clusters by comparing two persons from two randomly chosen different clusters. It is computed using the formula: $MOR=\exp \left(\sqrt{2\ast {V}_A\ast 0.6745}\ \right)\approx \exp \left(0.95\sqrt{V_A}\ \right)$, where *V*_*A*_ is the cluster level variance [10, 11]. The MOR measure is always greater than or equal to 1. If the *MOR* is 1, there is no variation between clusters [12].

### Model fit statistics

Deviance Information Criteria (DIC), Akaike’s Information Criterion (AIC), and Bayesian’s Information Criterion (BIC) is used to compare the candidate models. The model with the smallest value of the information criterion will be selected as the final model of the analysis [13].

## Result

### Socio-demographic characteristics

A total of 9450 sexually active rural women have participated in the EDHS-2016 survey. Regarding the age distribution, it was realized that about (33.5%) of those were youth (20 to 29) years. About 43.2% were Muslims followed by Orthodox Christian (33.9%). The most (67.5%) of women were married, followed by never married (23.0%). About half (55.0%) were poor, 29.9% were rich, while; others were middle wealth index status (19.0%). About the educational level, 58.6% of women and 70.0% of their husbands were not attending formal education. The majority of the women were housewives (70.8%). The age at first birth greater than seventeen years were (69.0%) (Table 1).

**Table 1:** Socio-demographic characteristics of sexual active rural women in Ethiopia

Variables	Categories	Frequency	Percentage
Contraceptive use	No	7474	79.1
	Yes	1976	20.9
Ever had a terminated pregnancy	No	8738	92.5
	Yes	712	7.5
Women occupation	Housewife	6693	70.8
	Government employed /farmer/private business	2757	29.2
Religion	Orthodox	3203	33.9
	Muslim	4087	43.2
	Others	2160	22.9
Age of women	10-19	2160	22.9
	20-29	3166	33.5
	30-39	2555	27.0
	40-49	1569	16.6
Education level of women	No education	5535	58.6
	Primary	3067	32.5
	Secondary and plus	848	9.0
Marital status	Never married	2106	22.3
	Married	6378	67.5
	Others	966	10.2
Number of living child	None	2729	28.9
	1-4	4051	42.9
	5 and plus	2670	28.3
Husband occupation	No	3798	40.2
	Yes	5652	59.8
Age of at first birth	< 17 year	2926	31.0
	> 17 year	6524	69.0
Wealth index	Poor	5200	55.0
	Middle	1798	19.0
	Riche	2452	25.9
Education of husband	No education	6612	70.0
	Primary	2119	22.4
	Secondary and plus	719	7.6
Births in last three years	No birth	5480	58.0
	One birth	3381	35.8
	Two and plus	589	6.2
Access to mass media	No	7975	84.4
	Yes	1475	15.6
	Tigray	1195	12.6
	Afar	816	8.6
Region	Amhara	1381	14.6
	Oromia	1498	15.9
	Somali	914	9.7
	Benishangul-Gumuz	902	9.5
	SNNPR	1495	15.8
	Gambela	643	6.8
	Harari	339	3.6
	Dire dawa	267	2.8

### The prevalence of modern contraceptive in sexually active rural women

**Fig. 1:**
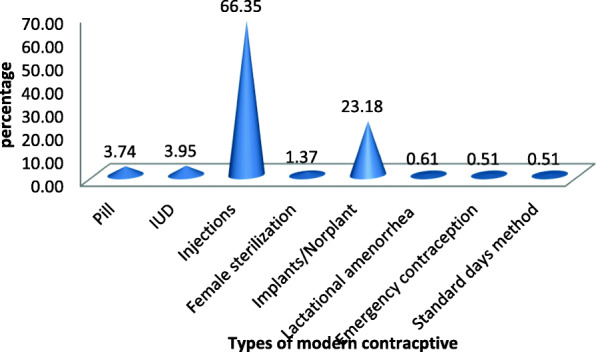
Usage of modern contraceptive method by type in rural women, Ethiopia

From a total of 9450 sexually active rural women, only 1976 (20.9%) were using a modern contraceptive method and the rest of 7474 (79.1%) did not use any modern contraceptive method. Among 1976 modern contraceptive users, 66.35% used injections, 23.18% used implant and the remaining 10.47% of women used the pill, IUD, female sterilization, emergency contraception, standard day method, and lactation amenorrhea method (Table 1 and Fig. 1).

### Predictors of modern contraceptive use among sexually active rural women in Ethiopia

The results of the multilevel logistic model both individual and community-level variables were summarized in Table 2. The model comparison result revealed that model IV is a better fit for the data as compared to other reduced models, since it has the smallest AIC and deviance statistic. In this model all individual-level and community-level factors are included, wealth index, educational level of the mother, education level of husband, age of women, occupation of women, religion, age at first birth, contraceptive use, number of living children, birth in the last three years, marital status, occupation of husbands, region, and access to mass media were factors significantly associated with modern contraceptive use (Table 2).

**Table 2:** Multilevel logistic regression output for predictor of modern contraceptive use among sexually active rural women in Ethiopia, 2016 EDHS

Variables	Model 1	Model 2	Model 3	Model 4
	AOR (95% CI)	AOR (95% CI)	AOR(95%CI)	AOR (95% CI)
**Individual-level factors**				
**Education of women**				
No education		1		1
Primary		1.03(0.92-1.14)		1.31(1.14-1.51)^a^
Secondary and plus		0.90(0.76-1.09)		1.39(1.06-1.81)^a^
**Occupation of respondents**				
Housewife		1		1
Government employed/farmer/private business		1.46(1.31-1.62)^a^		1.19(1.05-1.35)^a^
**Marital status**				
Never married		1		1
Married		27.82(19.32-40.06)^a^		17.31(10.72-27.94)^a^
Others		9.54(6.34-14.35)^a^		8.10(5.11-12.82)^a^
**Wealth index**				
Poor		1		1
Middle		2.24(1.97-2.55)^a^		2.05(1.77-2.38)^a^
Riche		2.55(2.27-2.87)^a^		2.26(1.96-2.60)^a^
**Age of respondents**				
10-19		1		1
20-29		5.03(4.18-6.06)^a^		1.14(0.89-1.46)
30-39		5.11(4.23-6.18)^a^		0.80(0.60-1.06)
40-49		3.17(2.57-3.91)^a^		0.40(0.29-0.56)^a^
**Religion**				
Orthodox		1		1
Muslim		0.29(0.26-0.33)^a^		0.29(0.25-0.33)^a^
Others		0.75(0.66-0.85)^a^		0.67(0.58-0.77)^a^
**Ever had a terminated pregnancy**				
No		1		1
Yes		1.15(0.96-1.38)		0.75(0.61-0.91)
**Number of living child**				
None		1		1
1-4		6.36(5.38-7.53)^a^		2.70(2.07-3.53)^a^
5 and plus		4.22(3.52-5.05)^a^		2.49(1.81-3.43)^a^
**Education of husbands**				
No education		1		1
Primary		3.72(3.32-4.16)^a^		1.45(1.27-1.67)^a^
Secondary and plus		3.18(2.69-3.77)^a^		1.02(0.82-1.27)^a^
**Husbands occupation**				
No		1		1
Yes		6.75(5.85-7.79)^a^		2.02(1.63-2.52)^a^
**Births in last three years**				
No birth		1		1
One birth		1.54(1.40-1.72)^a^		0.51(0.44-0.59)^a^
Two and plus		0.64(0.50-0.82)^a^		0.25(0.19-0.34)^a^
**Age of at first birth (years**)				
< 17		1		1
> 17		0.64(0.57-0.71)^a^		1.05(0.93-1.19)^a^
**Community-level factors**				
**Accesses to mass media**				
No			1	1
Yes			1.75(1.55-1.99)^a^	1.35(1.16-1.57)^a^
Region				
Tigray			1	1
Afar			0.06(0.04,0.10)^a^	0.12(0.07,0.20)^a^
Amhara			1.77(1.48,2.11)^a^	1.88(1.54,2.31)^a^
Oromia			0.86(0.71,1.03)	0.93(0.73,1.18)
Somali			0.02(0.01,0.04)^a^	0.04(0.02,0.10)^a^
Benishangul-Gumuz			0.80(0.65,0.99)^a^	0.83(0.64,1.07)
SNNPR			1.19(1.00,1.43)	1.45(1.13,1.87)^a^
Gambela			0.69(0.54,0.87)^a^	0.75(0.55,1.02)
Harari			0.41(0.29,0.58)^a^	0.43(0.28,0.65)^a^
Dire dawa			0.36(0.24,0.54)^a^	0.58(0.37,0.91)^a^

1 = reference category of the categorical variable; ^a^ Significant at 5% level of significance.

### Individual level factors

Attending the primary educational level was 1.31 (AOR = 1.31, 95%CI: 1.22, 1.51) times more likely to use modern contraceptives compared to not attending any formal education. Similarly, respondents with secondary and above educational level were 1.39 (AOR = 1.39, 95%CI: 1.06, 2.81) times more likely to use modern contraceptives compared to not attending any formal education. Additionally, women who had wrought in government-employed/farmer/business were 1.19 (AOR = 1.19, 95%CI: 1.05, 1.35) times more probable to use modern contraceptives than those who are housewives. Respondents aged 40-49 years were 0.40 (AOR = 0.29, 95%CI: 0.56, 2.81) times less likely to use modern contraceptives compared to 10-19 years old. Respondents having one to four living children were 2.70 (AOR = 2.70, 95%CI: 2.07, 3.53) times more likely to use modern contraceptives compared to respondents who had no living children. Respondents who had five or more living children were 2.49 (AOR = 2.49, 95%CI: 1.81, 3.43) times more likely to use modern contraceptives compared to respondents who had no living children. The likelihood of being willing to use modern contraceptives among Muslim respondents was 0.29 (AOR = 0.29, 95%CI: 0.25, 0.33) times less likely than that for Orthodox Christian respondents. Respondents in the middle wealth index status were 2.05 (AOR = 2.05, 95%CI: 1.77, 2.38) times more likely to use modern contraceptive than those respondents with poor wealth index status. Similarly, women with a rich wealth index were 2.26 (AOR = 2.26, 95%CI: 1.96, 2.60) times more likely to use modern contraceptives than women with a poor wealth index. Married women were 17.31 (AOR = 17.31, 95%CI: 10.72, 27.94) times more likely to use modern contraceptive than those never-married women. Women whose husbands attended primary education were 1.45 (AOR = 1.45, 95%CI: 1.27, 1.67) times more likely to use modern contraceptives than those who did not attend any formal education. Besides, women whose husbands had wrought with government-employed/farmer/private business were 2.02 (AOR = 2.26, 95%CI: 1.96, 2.60) times more probable to use modern contraceptives than those who had not worked. Women who gave birth to a child in the last three years were 0.51 (AOR = 0.51, 95%CI: 0.44, 0.51) times less likely to use modern contraceptives than those women not giving birth. Similarly, Women given birth to two and more children in the last three years were 0.25 (AOR = 0.25, 95%CI: 0.19, 0.34) times less likely to use modern contraceptive than those women not given birth (Table 2).

### Community-level factors

Respondents who have got access to mass-media were 1.35 (AOR = 1.35, 95%CI: 1.16, 1.57) times more likely to use modern contraceptive than who had not got any mass-media. Women living in Afar (AOR =0.12: 95%CI: 0.07, 0.20), Somali (AOR =0.04; 95% CI: 0.02, 0.10), Harari (AOR = 0.43; 95%CI: 0.28, 0.65), Dire Dawa (AOR = 0.58; 95%CI: 0.37, 0.91); regional state were less likely to use modern contraceptive as compared to women living in Tigray region (Table 2). However, Women living in Amhara (AOR 1.88; 95%CI: 1.54, 2.31) and SNNPR (AOR = 1.45; 95%CI: 1.13, 1.87) were more likely to use modern contraceptive as compared to women living in the Tigray region (Table 2).

### Random effect measures of variation

The results of random effects indicated that there was a statistically significant variation in the use of modern contraceptives across the clusters (Table 3). In other words, the use of modern contraceptives was not similarly distributed across the clusters. The Intra-cluster correlation coefficients (ICC) revealed that 31.64% of the variation in the use of modern contraceptives could be attributed to community-level factors. After adjusting for individual-level and community-level factors, there is a significant variation in the use of modern contraceptives across communities or clusters. About 86.69% of modern contraceptive use in clusters was explained in the full model. Moreover, the MOR confirmed that the use of modern contraceptives was attributed to community-level factors. The MOR for modern contraceptive use was 7.06 in the null model; this indicated that there was variation between communities (clustering), since; 7.06 times higher than the reference (MOR = 1). The unexplained community variation in the use of modern contraceptives was reduced to MOR of 2.04 when all factors were added to the model. This indicated that when all factors are considered, the effects of clustering are still statistically significant in the full models (Table 3).

**Table 3:** Measure of variation on individual and community level predictors among sexually active rural women in Ethiopia, EDHS-2016

Measure of variation	Model 1 (Null model)	Model 2	Model 3	Model 4 (Full model)
Variance (SE)	4.23(0.03)*	1.52 (0.07)*	3.09 (0.05)*	0.56(0.07)*
PCV (%)	Reference	64.00	27.07	86.69
ICC (%)	56.25	31.64	48.39	14.61
MOR	7.06	3.23	5.30	2.04
**Model fit statistics**				
DIC (−2log likelihood)	8951.65	7162.19	8362.95	6987.24
AIC	8955.65	7212.19	8384.95	7057.24
BIC	8969.96	7389.48	8463.64	7305.44

*reference *P*-value < 0.0001

## Discussion

Girls’ and women’s ability to control their fertility, and to decide if and when to have children and how many children to have, is the bedrock of women’s empowerment, gender equality, and progress for all. Therefore, this study was aimed to assess the prevalence of modern contraceptive use and associated predictors among sexually active rural women in Ethiopia.

The result of this study showed that 20.9% of the respondent used a modern contraceptive method which is lower than that reported from 2016 EDHS 35% [7]. In urban areas, women may have greater faith in decision-making confidence, autonomy, availability of contraceptive methods, and even better living standards than rural women [14]. One of the barriers was the longer distance to health facilities in rural areas, with more job opportunities in urban areas. And also women living in urban areas have good access like family planning to various services [15]. Similarly, this result was lower than Ghana(33.2%) [16], South Africa (41.8%) [16], and Kenya(68.9%) [17]. This might be due to the variations in socio-demographic features and the time gap between the studies.

Among modern contraceptive users, 66.35% of women use injection, 23.18% uses the implant, and 10.47% of women use other modern contraceptive methods. It coincides with studies done in Ethiopia [18, 19]. These findings have demonstrated women’s preference for short-acting hormonal contraceptive methods such as injections and tablets. However, in pregnancy prevention, long-acting reversible techniques such as IUDs and implants as well as permanent techniques are considered to be more effective [20].

The results of multilevel logistic regression for random effects indicated that there was a significant variation in the use of modern contraceptive methods across the clusters. In the last full model with both individual and community-level factors accounted for, about 86.69% of the variation was observed for the use of modern contraceptive methods. It was in agreement with studies in Ethiopia [14, 21], and Nigeria [22].

This study revealed that an increasing educational level of respondents; whose husbands were more likely to use a modern contraceptive method. This result was consistent with previous studies [14, 21–25]. This could be explained by the fact that educated women have better access to health care information, have greater autonomy to make decisions, and have a greater ability to use quality health care services. Women between the ages of 25-29 were more likely to use modern methods of contraceptive as compared to older women. This might be since younger women, who are married are more likely to do their first child to become pregnant [26]. Younger women can also have trouble obtaining family planning services because they may not know where contraception could be accessed or cannot afford services [27]. This association is also proofed in other studies across the world [28].

Several living children had shown a significant association with the use of modern contraceptives. The number of living children increased, the odds of using modern contraceptives are also increased. Previous studies also confirmed that a high number of living children were more likely to use modern contraceptives [14, 22, 24, 26, 29, 30]. The reason behind this might be due to that women might not want to have contraception before they have 3-4 children, after that, they want no more children and want to opt for family planning services. This finding suggests women will practice contraception when they meet their desired family size.

The wealth index was an important predictor of modern contraceptive use. Rich women were more likely to use modern contraceptives than poor women. Previous studies also showed that women in higher socio-economic families; more likely to use modern contraceptives [14, 22, 24, 26, 29, 31]. Women from wealthy households are more vulnerable to the media and more likely to use modern contraceptives [32]. This is mainly because rich people have access to more things, are better educated, and the capacity to make their own decisions.

Women’s employment status has also been associated with the use of modern contraceptives. Working women were more likely to use modern contraceptives than women who were housewives. It was consistent with previous studies [14, 22, 25, 31, 33, 34]. Women who have been working in different occupations are more likely than housewives to exchange knowledge and experience with their colleagues about modern contraception. It was also found that the level of media exposure had a positive association with modern contraceptive use. Women exposed to the media were more likely to use the modern method of contraception than women who were not exposed to the media. The finding is in line with [14, 21, 24, 29, 31]. This is because mass media exposure could be an important means of improving awareness and motivating women to practice some sort of modern contraceptives. Women, who had given birth to a child in the last 3 years were less likely to use modern contraceptive than those women who had not given birth. This result is in agreement with [14].

The result of this study indicated that religion was strongly associated with modern contraceptive use. Orthodox Christian women were more likely to use modern contraceptives than Muslims and others (Catholic, Protestant, and Traditional). This finding is in line with [21, 26, 31, 35]. This might be since the strong belief in the Muslim community in a holly book that considers family planning to be prohibited [26]. The attribute of this result needs further investigation. Never married women had lower odds of using modern contraceptives than those married and others (windowed and divorced). This result is in agreement with previous findings [25, 28, 34]. This is due to rural unmarried women might not practice sexual intercourse before marred. Besides, women whose husbands had working with government-employed/farmer/private businesses were more likely to use modern contraceptives than those who had not worked. The finding is in line with [14].

Furthermore, the use of modern contraceptives has often varied across regions. Women living in Afar, Somali, Harari, Dire Dawa region had lower use of modern contraceptive methods as compared to the Tigray region (*as a reference region*). This finding proofed studies in Ethiopia [14] and Malawi [27]. The potential explanation for this regional disparity is that there are variations between regions; in the implementation of family planning services. The non-accessibility of contraceptive methods leads to the highest under-five mortality in Ethiopia [34, 35]. This implies accessibility of contraceptive methods to use will reduce the mortality of children and further health complexity among mothers.

## Conclusion

The prevalence of modern contraceptive use in rural Ethiopia was 20%, which was very low. Injection (66.35%) was the most common type of modern contraceptive used. Educated women; educated husband, employed women, employed husband, married women, Orthodox Christian women, and at least one living child were positively associated with modern contraceptive use among individual-level factors. Access to media exposure, and regions were significantly associated with community-level factors with modern contraceptive use. Therefore, the government and other stakeholders shall provide educational opportunities; creating awareness about using modern contraceptives, and providing valuable counseling services. Those might increase modern contraceptive methods utilization.

## Data Availability

The survey datasets used in this study was based on a publicly available dataset that is freely available online with no participant’s identity from http://www.dhsprogram.com/data/available-datasets.cfm. Approval was sought from MEASURE DHS/ICF International and permission was granted for this use.

## Abbreviations

AIC: Akaike’s information criterion
AOR: Adjusted odds ratio
CI: Confidence intervals
DIC: Deviance information criterion
EAs: Enumeration areas
EDHS: Ethiopian demographic and health survey
FP: Family Planning
ICC: Intra-cluster correlation
MOR: Median odds ratio
PCV: Proportional change in variance
SNNPR: Southern Nations, Nationalities, and People Region
